# Commentary: 20-Year Trends in the Pharmacologic Treatment of Bipolar Disorder by Psychiatrists in Outpatient Care Settings

**DOI:** 10.3389/fpsyt.2020.592593

**Published:** 2021-01-15

**Authors:** Bruno Etain, Emilie Olie, Philippe Courtet, Ophelia Godin, Marion Leboyer

**Affiliations:** ^1^Université de Paris – INSERM UMRS 1144, Paris, France; ^2^Assistance Publique des Hôpitaux de Paris, APHP.Nord, DMU Neurosciences, GHU Saint Louis-Lariboisière-Fernand Widal, Département de Psychiatrie et de Médecine Addictologique, Paris, France; ^3^Fondation Fondamental, Créteil, France; ^4^Department of Emergency Psychiatry and Acute Care, Lapeyronie Hospital, PSNREC, Univ Montpellier, INSERM, CHU de Montpellier, Montpellier, France; ^5^Université Paris Est Créteil, Inserm U955, IMRB, Laboratoire Neuro-Psychiatrie Translationnelle, Créteil, France

**Keywords:** bipolar disorder, antipsychotics, suicide, lithium, metabolic syndrome

## Introduction

Using data from the US care system over 20 years, Rhee and colleagues (1) reported in the American Journal of Psychiatry (July 2020) that, among outpatients with Bipolar Disorder (BD), the prescriptions of second-generation antipsychotics (SGAs) massively increased (from 12.4% of BD outpatient in the 1997–2000 period to 51.4% in the 2013–2016 period), while the prescriptions of lithium and anticonvulsants decreased (from 62.3% of BD patients in the 1997–2000 period to 26.4% in the 2013–2016 period). The use of antidepressants, whose efficacy in bipolar depression is limited (2), constantly rose during the same period, these being often prescribed without any mood stabilizer (40.9% in the 2013–2016 period) in about half of the visits.

## The Growing Use of Second Generation Antipsychotics is Questionable

This shift from lithium/anticonvulsants to SGAs prescriptions is also observed in Europe (3, 4). This trend was expected because most SGAs have been marketed during the last two decades. RCTs published by drug companies made SGAs mechanically going up to the top of the list of recommended medications in recent guidelines. While formally approved in BD, the rising use of SGAs is nonetheless alarming as it is associated with metabolic syndrome (5). Metabolic syndrome, which is associated with a 2-fold higher rate of cardiovascular outcomes and a 1.5-fold higher all-cause mortality rate, affects 37% of patients with BD (6). Efforts are therefore required to bend this curve of SGAs prescriptions to prevent any potential long-term harm.

The decreasing use of lithium would certainly be viewed as a non-sense by many clinicians. Beyond its proven efficacy as a mood stabilizer, lithium is also known to reduce suicidal risk in BD (7). About one-third to one-half of BD patients attempt suicide at least once in their lifetime and approximately 15–20% die due to suicide. Not only recommended for the prevention of mood recurrences, lithium also decreases suicidal risk in BD, a striking advantage for a disorder for which the burden of suicide is major. Experts in the field regularly advocate for a renewal of lithium use despite its observed decline. Although it is undoubtedly easier to prescribe SGAs as first line agents (however, just a short-term option), clinicians should beware of the belief that SGAs necessarily provide a better benefit/risk balance. Hence, we should relentlessly argue that lithium offers an efficacious and safe option in BD which helps reduce the high rates of mortality due to suicide and natural causes.

The choice of medication has thus an impact on premature mortality in BD patients largely due to cardiovascular disease and suicide (8).

## Discussion

A decade ago, the “Fondamental Foundation” created the French Network of Centers of Expertise which offers recommendations for personalized care plans in BD. During a 2 years follow-up, we observed significant changes in the medications being prescribed [i.e., higher use of lithium or anticonvulsants, lower use of antidepressants, and in parallel, an improvement of the course of BD (i.e. mood episodes, hospitalizations) (9)]. We suggest that such centers can help guaranteeing a rationale and personalized use of medications in BD (personalized choice of mood stabilizers and a cautious use of antidepressants), in agreement with guidelines. Expertise should counterbalance common practices.

## Author Contributions

BE and EO wrote the draft of the manuscript. OG, PC, and ML actively revised the manuscript. All authors contributed to the article and approved the submitted version.

## Conflict of Interest

The authors declare that the research was conducted in the absence of any commercial or financial relationships that could be construed as a potential conflict of interest.
